# Retinal ischemia-induced apoptosis is associated with alteration in Bax and Bcl-x_L_ expression rather than modifications in Bak and Bcl-2

**Published:** 2009-10-19

**Authors:** Nathalie Produit-Zengaffinen, Constantin J. Pournaras, Daniel F. Schorderet

**Affiliations:** 1 Institute for Research in Ophthalmology, Sion, Switzerland; 2 Department of Ophthalmology, Geneva University Hospitals, Geneva, Switzerland; 3 Department of Ophthalmology, University of Lausanne, Lausanne, Switzerland; 4 Institute of Life Sciences, Federal Polytechnic School of Lausanne, Lausanne, Switzerland

## Abstract

**Purpose:**

Apoptosis is known to play a key role in cell death after retinal ischemia. However, little is known about the kinetics of the signaling pathways involved and their contribution to this process. The aim of this study was to determine whether changes in the expression of molecules in the mitochondrial apoptotic pathway might explain the progression of retinal damage following ischemia/reperfusion.

**Methods:**

Retinal ischemia was induced by elevating intraocular pressure in the vitreous cavity to 150 mmHg for a period of 60 min. At time 0, 3 h (early phase), and 24 h (late phase) after reperfusion, the retinas were harvested and modifications in the expression of Bax, Bak, Bcl-2, and Bcl-x_L_ as well as caspase-3 and −7, were examined by qPCR and, in some cases, by western blot.

**Results:**

qPCR analysis performed at the early phase after ischemia revealed a time dependent decrease in *Bax, Bak*, and *Bcl-x_L_* and no alteration in *Bcl-2* mRNA expression in response to retinal ischemia. At the protein level, proapoptotic Bax and Bak were not modulated while Bcl-2 and Bcl-x_L_ were significantly upregulated. At this stage, the Bax per Bcl-2 and Bax:Bcl-x_L_ ratios were not modified. At the late phase of recovery, *Bax* and *Bcl-x_L_* mRNAs were downregulated while *Bak* was increased. Increased Bax:Bcl-2 and Bax:Bcl-x_L_ ratios at both the mRNA and protein levels were observed 24 h after the ischemic insult. Analysis of caspases associated with mitochondria-mediated apoptosis revealed a specific increase in the expression of *caspase-3* in the ischemic retinas 24 h after reperfusion, and a decrease in the expression of *caspase-7*.

**Conclusions:**

This study revealed that Bcl-2-related family members were differently regulated in the early and late phases after an ischemic insult. We showed that the Bax:Bcl-2 and Bax:Bcl-x_L_ balances were not affected in the initial phases, but the Bax:Bcl-x_L_ ratio shifted toward apoptosis during the late phase of recovery. This shift was reinforced by *caspase-3* upregulation.

## Introduction

Retinal cell death induced by transient ischemia occurs through necrosis and apoptosis. The latter has been widely studied. TUNEL staining has revealed that apoptotic cells can be observed as early as 3 h after the ischemic insult and that their number progressively increased and reached a plateau at 24 h [1-3]. These cells are mainly located in the ganglion cell layer, the inner nuclear layer, and, to a lesser extent, the outer nuclear layer [1,2]. In the case of moderate retinal ischemia, as in experimental branch vein occlusion, apoptosis has also been observed at peripheral retinal areas and has been associated with diffuse edema of the inner nuclear layer [4].

The mechanisms underlying apoptosis following ischemia/reperfusion (I/R) have been extensively studied. Special attention has been given to the mitochondria-mediated apoptotic pathways, in particular to the Bcl-2 family members, which are important regulators of cell death in the brain, heart, liver, kidney, and retina [5-9]. This family of proteins is divided into proapoptotic members, including Bax, Bak, and Bcl-2 homology domain 3 (BH3)-only proteins (i.e., Bad, Puma, and Noxa) and antiapoptotic members, such as Bcl-2 and Bcl-x_L_. In a nonapoptotic environment, Bax is maintained in the cytosol, sequestered by the antiapoptotic Bcl-2 and Bcl-x_L_ [10]. When stimulated, Bax is activated and translocated to the mitochondria, where it forms pores in the outer membrane by binding with the adenine nucleotide translocator leading to permeabilization of the mitochondria [11-13]. This in turn initiates cytochrome C release from the mitochondria and activates the Apaf-1–caspase-9 apoptosome and downstream effector caspases [14].

Increased *Bax* and decreased *Bcl-2* expressions have been described in several models of cerebral ischemia, and a reduction in antiapoptotic *Bcl-x_L_* mRNA following traumatic brain injury has also been reported [15-18]. These studies suggest that an imbalance in the regulation between pro- and antiapoptotic molecules triggers apoptosis. Similar studies were performed on the retina but, excepting the consensus observation that *Bax* expression was upregulated following an ischemic insult, conflicting results were obtained for *Bcl-2* regulation [1,19-21].

Clarification of the mechanisms involved in retinal ischemia, and a better understanding of potential similarities between brain and retinal injury following an ischemic insult, would serve to accelerate the development of therapies. The aim of this study is, therefore, to unravel the sequential modulation of members of the Bcl-2 family following I/R in the retina.

## Methods

### Animal handling and surgery

All experimental procedures involving rats were performed in accordance with the ARVO Statement for the Use of Animals in Ophthalmic and Vision Research and were approved by the local Committee Office on Use and Care of Animals in Research of the State of Valais, Sion, Switzerland.

The procedure to induce transient ischemia followed by reperfusion has already been described [22]. In brief, 250–300 g male Sprague-Dawley rats, purchased from Janvier (Le Genest-St-Isle, France), were first anesthetized by isoflurane inhalation and then by intraperitoneal injection of a freshly made mixture of 100 mg/kg ketamine hydrochloride and 10 mg/kg xylazine hydrochloride. Rat pupils were dilated with 0.5% atropine drops, and corneas were anesthetized with 2 drops of 0.4% oxybuprocain. During the whole procedure, rats temperature was monitored and maintained at 37 °C. A steel 30-gauge infusion needle (Becton Dickinson, Basel, Switzerland) connected to a saline reservoir was inserted into the vitreous cavity of the left eye. The pressure was elevated to 150 mmHg for a period of 1 h. Retinal and choroidal bloodstream arrest was immediately observed under the binocular microscope Stemmi 2000-C (Zeiss, Feldbach, Switzerland). After 60 min of ischemia, the pressure was restored to normal levels by removing the needle and the perfusion immediately resumed. The integrity of the retinal blood flow was observed with the operating binocular microscope both before and after the procedure.

Animals were divided into a control and an I/R group. The control group was divided into two subgroups: anesthesia only; and sham-operated, which was done by inserting a needle into the vitreous cavity of the rat’s left eye without causing elevation of the intraocular pressure (IOP). In the I/R group, the needle was inserted into the vitreous cavity of the left eye and the pressure increased. At 3 or 24 h after reperfusion the rats were anesthetized with isoflurane and euthanized with an intraperitoneal administration of an overdose of 150 mg/kg bodyweight sodium pentobarbital (Escarnakon; Streuli Pharma SA, Uznach, Switzerland). Both eyes were enucleated and the retinas carefully dissected.

### RNA isolation

The retinas were dissected under a binocular microscope to exclude extraretinal tissues, then rapidly isolated in RNAlater (Ambion; Applied Biosystems, Rotkreuz, Switzerland) before being transferred to TRIzol reagent (Invitrogen AG, Basel, Switzerland) and stored at −80 °C until RNA extraction. Total RNA was extracted following the manufacturer’s instructions. Both quantity and quality of RNA were determined on a ND-1000 spectrophotometer (NanoDrop technologies, Inc., Wilmington, DE).

### Quantitative reverse transcription-PCR

cDNA synthesis was performed using 2 μg of total RNA in 20 μl reaction volume. This was done using an oligo dT primer according to the manufacturer’s manual (Affinity Script; Stratagene; Agilent technologies SA, Morges, Switzerland). The equivalent of 12.5 ng of original total RNA was used for quantitative PCR amplification using the 2× brilliant SYBR Green QPCR Master Mix (Stratagene; Agilent technologies SA, Morges, Switzerland). One μM forward and reverse primers, designed to span an intron of the target gene were added to the mix. Table 1 gives the sequence of the primers used in this study. Real-time PCR was performed in a Mx3000PTM system (Stratagene) with the following cycling conditions: 40 cycles of denaturation at 95 °C for 30 s, annealing at 59 °C for 30 s, and extension at 72 °C for 30 s. Samples were amplified in triplicate, and data were normalized with expression of *β-actin*.

**Table 1:** Primers for quantitative PCR

**Gene (GenBank accession number)**	**Primers (5′-3′)**	**Size (bp)**
*Bax* (NM_017059)	F: GAGCGGCTGCTTGTCTGGAT	161
	R: CAAGGCAGCAGGAAGCCTCA	
*Bak* (NM_053812)	F: AATGGCATCCGGACAAGGAC	135
	R: TGTTCCTGCTGGTGGAGGTA	
*Bcl-2* (NM_016993)	F: CTGAACCGGCATCTGCACAC	195
	R: GCAGGTCTGCTGACCTCACT	
*Bcl-xL* (NM_001033670)	F: GGTCGCATTGTGGCCTTCTT	196
	R: CTCTCGGCTGCTGCATTGTT	
*Casp 3* (NM_012922)	F: GACTGCGGTATTGAGACAGA	209
	R: CGAGTGAGGATGTGCATGAA	
*Casp 7* (NM_022260)	F: CAACGACACCGACGCTAATC	161
	R: GGTCCTTGCCATGCTCATTC	
Actin-β (NM_031144)	F: AGGCCAACCGTGAAAAGATG	100
	R: ACCAGAGGCATACAGGGACAA	

This table gives accession number, primers sequence for quantitative PCR and amplicon size.

### Western blot analysis

The retinas were rapidly dissected in 1× phosphate-buffered saline (PBS; NaCl 137 mM, KCl 2.7 mM, Na_2_HPO_4_ 10 mM, KH_2_PO_4_ 1.75 mM, pH 7.4) supplemented with a 1× protease inhibitor cocktail (Roche Applied Science, Basel, Switzerland). Retinas were then transferred to a Hepes-Tween solution, which contained 20 mM Hepes and 0.5% Tween-20, and included 1× phosphatase and 1× protease inhibitor cocktails (Sigma-Aldrich, Basel, Switzerland), before they were stored at −80 °C until protein extraction. Samples were frozen and thawed three times, then homogenized on ice. Supernatant was collected after 30 min of centrifugation at 9.3 xg at 4 °C. The amount of protein was determined using a bicinchoninic acid (BCA) protein assay kit (Pierce Biotechnology, Rockford, IL). As loading control, β-actin was measured in each western blot. Protein samples were run on 12% SDS–PAGE and transferred to a polyvinylidene fluoride membrane (Whatman; Schleicher and Schuell, St. Marcel, France) for 1 h at 4 °C. Membranes were then blocked at room temperature for 1 h in 1× tris-buffered saline (TBS; NaCl 137 mM, TRIS 25 mM, pH 7.4) and 0.1% Tween-20 and incubated with the appropriate antibody overnight at 4 °C. Antibodies were diluted in 1× TBS and 0.1% Tween-20 containing 2% blocking reagent (ECL Advanced; Amersham Biosciences, Otelfingen, Switzerland). Next, 1:1,000 rabbit anti-Bcl-2 (#2876), 1:1,000 anti-Bcl-x_L_ (#2764), and 1:1,000 anti-Bak (#3814) were obtained from Cell Signaling Technology (Bioconcept, Alschwill, Switzerland), rabbit anti-Bax (#493) from Santa Cruz Biotechnology (LabForce AG, Nunnigen, Switzerland) and 1:10,000 mouse anti-β-actin (#A5441) from (Sigma-Aldrich, Basel, Switzerland). Detection was performed with horseradish peroxidase-conjugated secondary antibodies (Amersham Biosciences; Otelfingen, Switzerland) and visualized by enhanced chemiluminescence (Millipore Corporation, Billerica, MA,) on a luminescent image analyzer (LAS 4000mini; Bucher Biotech, Basel, Switzerland). Quantification analysis of the proteins was performed with Multigauge (Fujifilm) software.

### Statistics

Results are presented as mean ±standard error of the mean (SEM) of the indicated number of independent experiments. Statistical analysis was performed using the Student’s *t*-test. Differences were considered significant for p<0.05.

## Results

To unravel the mechanisms involved in the apoptotic process induced after retinal ischemia, we assessed whether members of the *Bcl-2* family of genes were regulated early (3 h) or late (24 h) after I/R, both at the mRNA and protein levels. As our preliminary experiments indicated that both anesthesia and sham-insertion of a needle in the vitreous cavity were associated with modulation of several of the genes investigated, we compared each measurement with values from sham-operated rats set to 1.

### Early modulation at mRNA level

**Figure 1:**
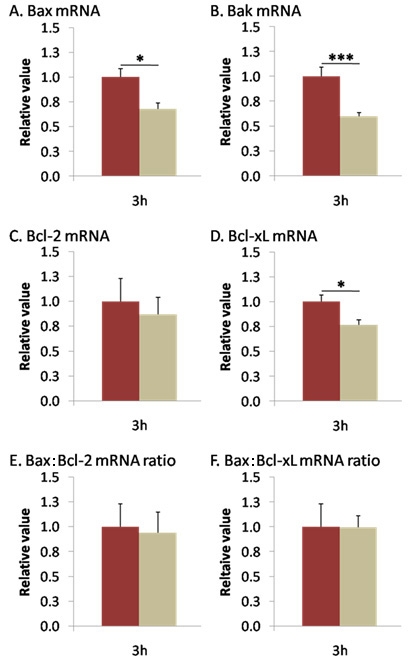
Ischemia reduced *Bax*, *Bak*, and Bcl-x_L_ mRNAs expression 3 h after I/R. *Bax* (**A**), *Bak* (**B**), *Bcl-2* (**C**), and *Bcl-x_L_* (**D**) mRNAs expression was measured by qPCR 3 h after reperfusion. At 3 h after reperfusion, ischemia decreased *Bax* (**A**), *Bak* (**B**), and *Bcl-x_L_* (**D**) expression but did not alter the *Bcl-2* mRNA level (**C**). **E** and **F** showed no modification in *Bax*:*Bcl-2* nor *Bax*:*Bcl-x_L_* mRNA ratios in the early phase after I/R. Brown columns denote control retinas (n=6), and beige columns denote ischemic retinas (n=10). Error bars represent SEM, where *p<0.05, ***p<0.001 in control versus ischemic retinas as measured by Student's *t*-test.

Three hours after I/R, the following proapoptotic *Bax* (0.679±0.058, p<0.05) and *Bak* mRNAs (0.600±0.033, p<0.001), as well as the antiapoptotic *Bcl-x_L_* (0.768±0.047, p<0.05) were significantly downregulated. The antiapoptotic *Bcl-2* did not change (0.868±0.169; Figure 1A-D). Rather than individual gene alteration, it is the ratio between pro- and antiapoptotic members that is responsible for cell fate. We therefore calculated the *Bax*:*Bcl2* and *Bax*:*Bcl-x_L_* ratios in each retina, but neither ratio showed any alteration 3 h after I/R (0.940±0.205 and 0.996±0.113, respectively; Figure 1E-F).

### Early modulation at protein level

**Figure 2:**
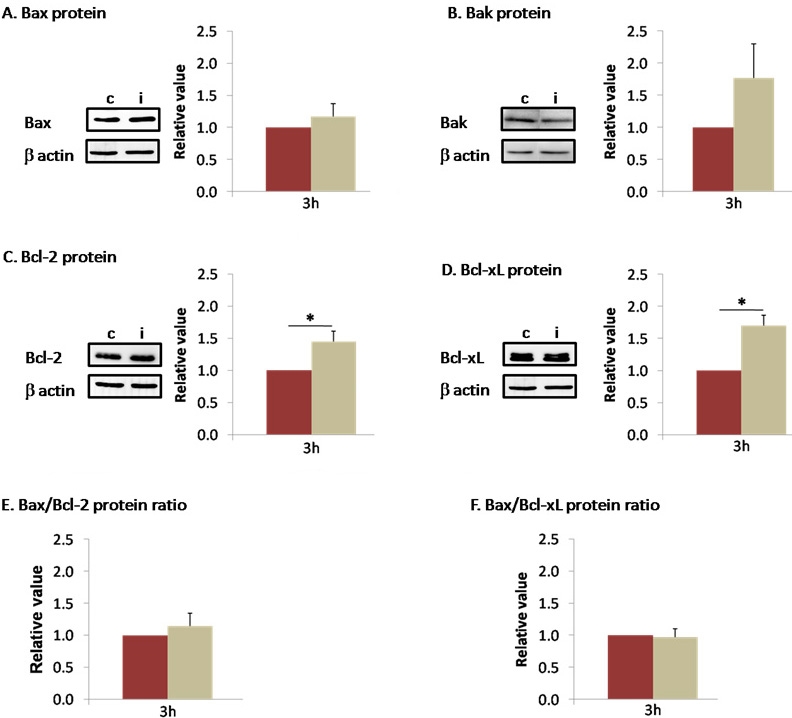
Ischemia did not modify proapoptotic but increased antiapoptotic proteins 3 h after I/R. Effect of ischemia 3 h after reperfusion on Bax (**A**), Bak (**B**), Bcl-2 (**C**), Bcl-x_L_ (**D**) protein levels measured by western blot. In (**E**-**F**) no modification in Bax/Bcl-2 nor Bax/Bcl-x_L_ protein ratios were observed at this time. Brown columns denote control retinas (n=6–10), and beige columns denote ischemic retinas (n=8–12). Error bars represent SEM, where *p<0.05 in control versus ischemic retinas as measured by Student's *t*-test.

At the protein level, Bcl-2 and Bcl-x_L_ increased 3 h after I/R (1.446±0.163, and 1.697±0.294, respectively) while Bax and Bak levels increased slightly but not significantly (1.171±0.136, 1.769±0.531; Figure 2A-D). As for mRNA, the Bax:Bcl-2 and Bax:Bcl-x_L_ protein ratios did not reveal any modulation (1.144±0.238 and 0.970±0.129, respectively; Figure 2E-F).

### Late modulation at mRNA level*:*

**Figure 3:**
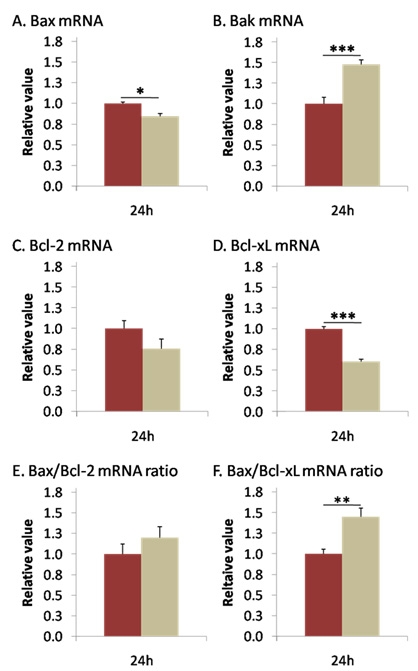
mRNA levels of *Bak* increased and *Bcl-x_L_* decreased 24 h after I/R. *Bax* (**A**), *Bak* (**B**), *Bcl-2* (**C**), and *Bcl-x_L_* (**D**) mRNAs expression was measured by qPCR 24 h after reperfusion. Twenty-four h after reperfusion, ischemia induced a slight decrease in *Bax* (**A**), and strongly increase *Bak* (**B**) mRNA expression. Anti-apoptotic *Bcl-2* expression (**C**) was not modified, but *Bcl-x_L_* (**D**) was strongly reduced 24 h after I/R. Figure 1 E,F revealed no modification in *Bax*:*Bcl-2* and a highly significant increase in *Bax*:*Bcl-x_L_* mRNAs ratio 24 h after I/R. Brown columns denote control retinas (n=6), and beige columns denote ischemic retinas (n=10). Error bars represent SEM, where *p<0.05, **p<0.005, ***p<0.001 in control versus ischemic retinas as measured by Student's *t*-test.

Analyses 24 h post I/R revealed that *Bax* and *Bcl-x_L_* were downregulated (0.841±0.033, p<0.05, and 0.604±0.025, p<0.01, respectively) while *Bak* was upregulated (1.478±0.052, p<0.001; Figure 3A-B,D). The strong decline of *Bcl-x_L_* was further amplified in the *Bax*:*Bcl-x_L_* ratio (1.446±0.106; p<0.005; Figure 3F) *Bcl-2* was not significantly modulated (0.759±0.116), nor was the *Bax*:*Bcl-2* ratio (1.194±0.132, Figure 3C,E).

### Late modulation at protein level

**Figure 4:**
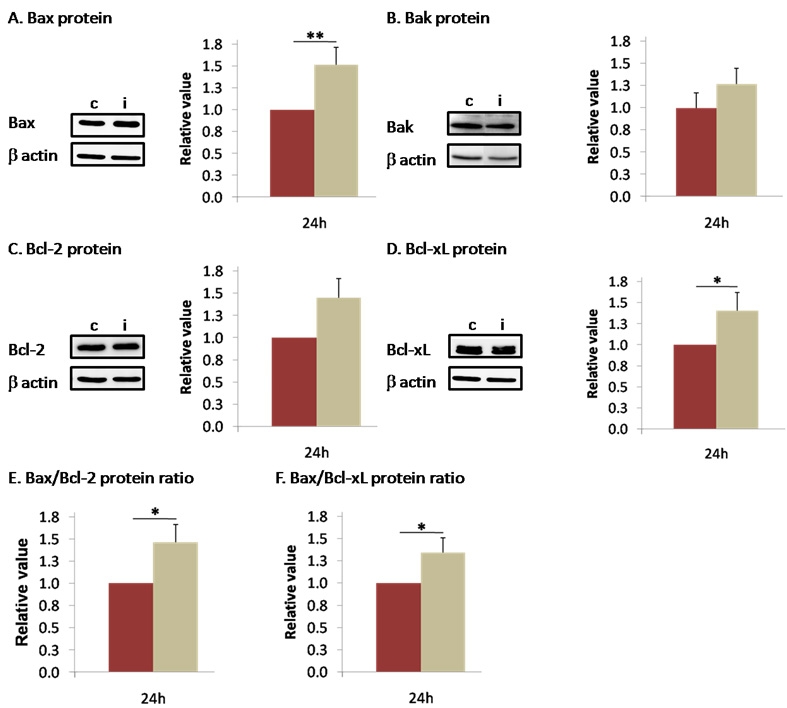
Ischemia induced a protein imbalance toward apoptosis 24 h after the ischemic insult. Bax (**A**), Bak (**B**), Bcl-2 (**C**), and Bcl-x_L_ (**D**) protein levels were measured by western blot 24 h after I/R. At this time proapoptotic Bax was significantly increased (**A**), but there was no modification in Bak (**B**). No modification in Bcl-2 (**C**) and an increase in Bcl-x_L_ (**D**) protein levels were observed 24 h after reperfusion. In **E** and **F**, a significant increase in Bax:Bcl-2 and Bax:Bcl-x_L_ protein ratios were measured at this time. Brown columns denote control retinas (n=6–10) and beige columns denote ischemic retinas (n=8–12). Error bars represent SEM, where *p<0.05, **p<0.005 in control versus ischemic retinas as measured by Student's *t*-test.

Contrary to mRNA, Bax and Bcl-x_L_ protein levels were significantly upregulated 24 h post I/R (1.511±0.148 and 1.402±0.188, p<0.05, respectively). The strong upregulation seen for *Bak* at the mRNA level was not present at the protein level (1.270±0.177). Bcl-2 protein did not change significantly (1.447±0.217). The shift of balance toward apoptosis was also evident on the Bax:Bcl-2 and Bax:Bcl-x_L_ ratios (1.463±0.216, 1.340±0.165; p<0.05; Figure 4A-F).

**Figure 5:**
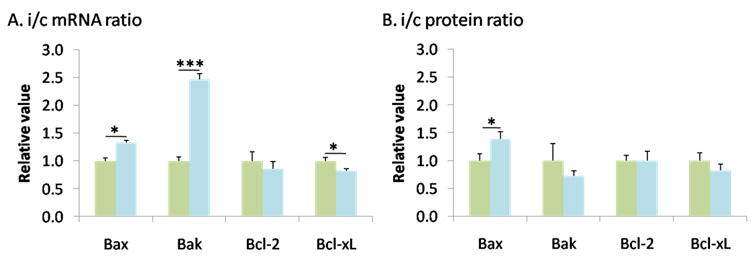
Bcl-2 family members are similarly regulated at the mRNA and protein levels. Ischemia modified Bax, Bak, Bcl-2, and Bcl-x_L_ mRNA (**A**) and protein expression (**B**) 24 h after reperfusion relative to 3 h. **A**: Proapototic *Bax* and *Bak* mRNAs expression was increased, whereas antiapoptotic *Bcl-x_L_* was decreased and *Bcl-2* expression unaltered 24 h after I/R. **B**: At the protein level, Bax expression was significantly increased but there was no modification in Bak, Bcl-2, or Bcl-x_L_ when measured 24 h after I/R. Green columns mark ischemic/ctrl ratio (i/c) ratio at 3 h, and light blue columns indicate i/c ratio at 24 h. Error bars represent SEM, where *p<0.05, ***p<0.001 in control versus ischemic retinas as measured by Student's *t*-test.

We then focused on the kinetics of the response and compared the modulation of the Bcl-2 family members between the early (3 h) and late (24 h) phases post I/R. Bax expression significantly increased by about 30% both at the mRNA (1.325±0.044) and protein levels (1.383±0.135), while Bcl-x_L_ decreased by about 20% at the mRNA (0.787±0.033) and protein levels (0.826±0.106). Bak was upregulated at the mRNA level (2.462±0.103) but this was not confirmed at the protein level (0.717±0.387). Here again, these seemingly small modifications of expression were further amplified when the Bax:Bcl-x_L_ ratio was analyzed, and a strong shift of the pro- and antiapoptotic balance toward apoptosis was observed. Interestingly, Bcl-2 did not seem to be involved in the kinetics of the response to I/R because it did not change significantly (Figure 5).

### Ischemia-induced *caspase-3* and repressed *caspase-7* mRNAs expression 24 h after I/R

**Figure 6:**
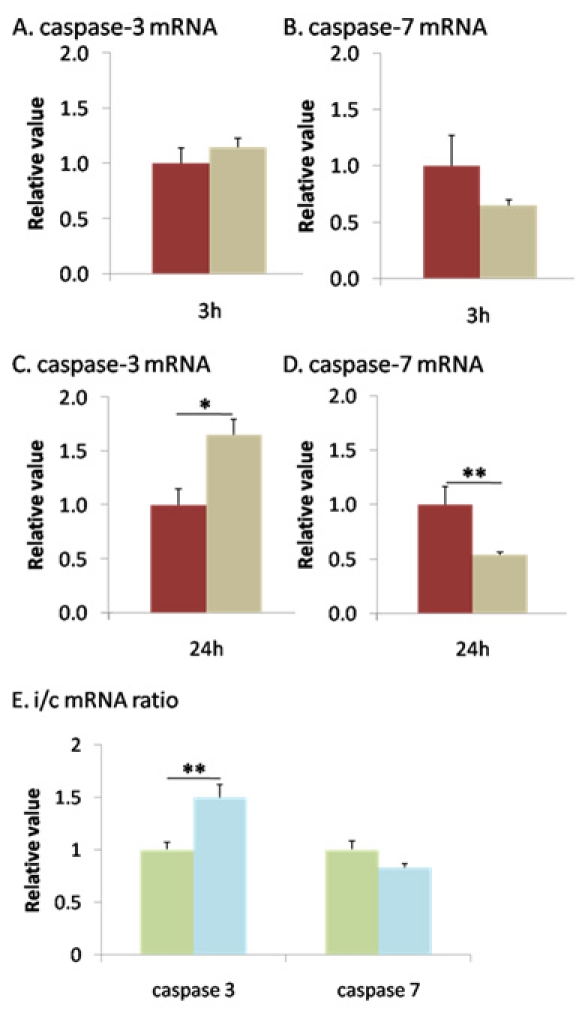
Ischemia induced *caspase-*3 and repressed *caspase-7* mRNAs expression 24 h after reperfusion. *Caspase-3* (**A**-**C**) and *caspase-7* (**B**-**D**) mRNAs expression was measured by qPCR 3 h (**A**-**B**) and 24 h (**C**-**D**) after reperfusion. **A**-**B**: No change in *caspase-3* (**A**) or *caspase-7* (**B**) was observed 3 h after I/R. **C**-**D**: Ischemia induced a significant increase in *caspase-3* (**C**) and decrease in *caspase-7* (**D**) mRNA 24 h following the ischemic insult. **E**: Ischemic/ctrl ratio (i/c) mRNA ratios reported an important increase in *caspase-3* and no changes in *caspase-7* between 3 and 24 h after I/R. Brown columns mark control retinas (n=6–10), and beige columns denote ischemic retinas (n=8–12). Green columns represent i/c ratio at 3 h, and light blue columns indicate i/c ratio at 24 h. Error bars represent SEM, where *p<0.05, **p<0.005 in control versus ischemic retinas, and #p<0.01 ischemic retinas at 3 h versus 24 h as measured by Student's *t*-test.

To further investigate the impact of retinal ischemia on downstream effectors of apoptosis stimulated by members of the Bcl-2 family, we analyzed the expression of *caspase-3* and *caspase-7* by qPCR. In the early recovering phase, *caspase-3* did not change (1.147±0.077) and *caspase-7* was downregulated (0.646±0.052). At 24 h post I/R, *caspase-7* was further reduced (0.536±0.025, p<0.005) while *caspase-3* was upregulated (1.656±0.141, p<0.05) (Figure 6A-D). Kinetics analysis of caspase regulation revealed a strong increase in *caspase-3* expression (1.494±0.128, p<0.005) and no modulation of *caspase-7* (0.830±0.039; Figure 6E-F).

## Discussion

Artificial IOP increase is a recognized model of retinal ischemia and is accompanied by apoptosis. We and other researchers have observed in this model that the number of dying cells increases from 3 h (early) until 24 h (late) after I/R [3]. In this study, we analyzed the response of several *Bcl-2*-related genes in an early and late period following I/R.

We first considered the regulation of several *Bcl-2* family genes at time 0, 3, and 24 h in control retinas from anesthetized animals and from anesthetized animals in which a needle had been inserted and kept in place for 1 h, but without initiating any increase in IOP. We observed that the sole insertion of a needle triggered upregulation of several genes. Therefore, and to specifically track the changes induced by ischemia rather than by anesthesia or needle insertion, we compared gene regulation between ischemic and needle inserted-only retinas. Our results showed a dysregulation between pro- and antiapoptotic genes resulting in a proapoptotic signaling cascade in ischemic retinas. This underlined the importance of a time-related upregulation of the proapoptotic Bax and did not support a relevant role for Bak in this deleterious process. The injury is mostly due to Bcl-x_L_ downregulation rather than downregulation in Bcl-2 expression. Although slight differences between mRNA and protein regulation were obtained in ischemic and control retinas, Bax:Bcl-2 and Bax:Bcl-x_L_ ratios were similarly modulated over time. Upregulation of Bax at the protein level is in agreement with in vitro and in vivo studies of retinal ischemia [1,19-21] as well as observations made after cerebral ischemia [15-17]. However, we demonstrated that Bax mRNA and protein increase is time dependent, suggesting a delayed role for this gene in the apoptotic process induced by I/R.

The regulation of Bcl-2 has widely been considered in the study of the retina as well as in cerebral ischemia. In the central nervous system, however, the role of Bcl-2 in response to neuronal injury is unclear because both increases and decreases in Bcl-2 levels have been described. In vitro, a decrease in the expression of Bcl-2 has been reported in cultured retinal cells stimulated by various means, including simulated ischemia and excitotoxicity [19], whereas no obvious change in *Bcl-2* gene expression was observed in vivo in retinas where ischemia was induced by increased IOP [1]. In another report, retinal ischemia was shown to induce an initial decrease in *Bcl-2* mRNA expression followed by a later increase in its expression [21]. The discrepancies of the results reported on *Bcl-2* expression are reinforced by the observation that Bcl-2 peptide administration did not increase retinal ganglion cell survival after optic nerve section but did so after selective ligature of the ophthalmic vessels [23]. Clearly, Bcl-2 involvement is not identical in these different models. In our study, we showed an increase of this protein during the early phase following I/R, after which its level remained constant until at least 24 h. This observation is in accordance with the results obtained by Kaneda et al. [1] at the mRNA level.

Bcl-2 has also been assessed in the experimental model of glaucoma after hypertonic saline injection [24]. In this study, the increase of apoptosis in retinal ganglion cells was linked to a downregulation of the *Bcl-2:Bax* ratio. However, this decrease was associated with a marked reduction in *Bcl-2* rather than with an increase in *Bax* itself [24]. The difference in the role of Bcl-2 in our study could result from the experimental model and from the duration of the experiment. Furthermore, our results do not exclude Bcl-2 downregulation after 24 h.

A mRNA study of *Bcl-x_L_* regulation after I/R revealed that it was slightly but significantly downregulated in comparison to sham-operated retinas. Consistent with mRNA and protein regulation was the time-dependent downregulation of Bcl-x_L_. Our results are in agreement with the moderate decrease in *Bcl-x_L_* mRNA expression in the retina observed after optic nerve crush [25]. The observation that *Bcl-x_L_* is the predominant Bcl-2 family member expressed in the retina supports the concept that the neural retina has a pattern of Bcl-2 expression similar to that of other central neurons [25,26]. This suggests that modification in Bcl-x_L_ expression after I/R in the retina could be identical to that following cerebral ischemia. However, our results showing a time-related decrease in Bcl-x_L_ expression in the retina following I/R are in conflict with a publication on hippocampal neurons, where no alteration of the abundance of Bcl-x_L_ was observed following I/R [27].

The observation that neither the Bax:Bcl-2 nor the Bax:Bcl-x_L_ ratios are regulated at a time when apoptosis is already detectable (3 h) suggests that Bcl-2-related family members are not associated with early cell death following I/R. However, the number of apoptotic cells may not be sufficient to allow for an accurate reading of the contribution of Bcl-2 family members. An alternative explanation could be that apoptosis is triggered not by an increase in Bax expression but by its translocation to the mitochondria, as has been demonstrated in a central retinal artery occlusion model of retinal ischemia [2]. More investigations regarding the activity of Bax are needed to attest Bcl-2 involvement in the deleterious effect induced by an ischemic insult of the retina.

Bak, another member of the Bcl-2 family, has been shown to change its intracellular location early in the promotion of apoptosis [28]. In comparison to *Bax*- or *Bak*-deficient mice, *Bax* and *Bak* double knockout mice displayed multiple developmental defects, including an accumulation of excess cells within the central nervous system. This indicated that both Bax and Bak have overlapping roles in the regulation of apoptosis during mammalian development and tissue homeostasis [29]. In the same way, *Bax* and *Bak* double knockout cells showed resistance to apoptosis induced by various stimuli, demonstrating the crucial role of these proteins in the regulation of cell death [11]. However, a 2006 study underlined the role of Bax and Bak in the regulation of mitochondrial dynamics in healthy cells and indicated that Bcl-2 family members may also regulate apoptosis through organelle morphogenesis [30]. Bax and Bak were also reported to act as essential components of the unfolded protein response. Although this role is independent of their proapoptotic function, it appeared that Bax and Bak might act as stress sentinels that connect stress signals to the proapoptotic core when cellular homeostasis is irreversibly altered [31].

As these different studies support a major role for Bak in the regulation of apoptosis, we also analyzed its expression by quantitative PCR and western blot. Surprisingly, our results showed that Bak regulation does not play a fundamental role in apoptosis following retinal ischemia. Indeed, even if its mRNA expression was time-dependently regulated, the observation that it was not altered by the ischemic insult at the protein level suggested that Bak level was not crucial to the apoptotic event following I/R at the time studied. However, the analysis of Bak expression later than 24 h after I/R could also reveal an important role of this protein. Furthermore, the absence of Bak protein regulation did not exclude that its apoptotic effect could be induced by its translocation rather than by its regulation after I/R, but this remains to be demonstrated [28].

Bax and Bak activation, opening of the mitochondrial permeability transition pore (mPTP), and cell death have been reported to be mediated through Bcl-2/adenovirus E1B 19 kDa protein-interacting protein 3 (Bnip3), a BH3-only protein localized primarily in mitochondria [32-34], although this is still controversial [35]. In a model of myocardial ischemia and reperfusion, Bnip3 was reported as a mitochondrial sensor of oxidative stress [36], thus contributing to cell death during I/R in ex vivo-perfused hearts and in cell culture [37,38]. Therefore, it would be interesting to study Bnip3-induced activation of Bax and Bak and to analyze whether Bnip-3 could also be associated with the increase in apoptosis observed after I/R in the retina. However, preliminary results more readily suggest a decrease in *Bnip-3* expression after I/R. Bellot et al. very recently demonstrated that hypoxia induced autophagy via Bnip-3 and that Bnip-3L was clearly a survival mechanism [39]. However, these observations were not in accordance with Puyal et al. [40], whose work revealed that cerebral postischemic autophagy inhibition was neuroprotective. Further studies are needed to resolve this issue and to determine the proapoptotic or prosurvival action of Bnip-3 expression.

We also showed that caspase mRNA was specifically modulated by ischemia, as we reported a time-related increase in *caspase-3* and decrease in *caspase-7*. Caspase-3, a well known apoptosis effector, has been studied in several models of retinal ischemia, including IOP elevation [41-43] and axotomy [44-46]. Caspase-7, which is structurally and functionally similar to caspase-3, has been shown to compensate for the lack of caspase-3 [47,48]. Therefore, it seems likely that the absence of caspase-7 upregulation is a consequence of the regulation of caspase-3.

In summary, the current study reports a novel regulation of Bcl-2-related family members following retinal ischemia. It clearly finds an association with an increase in Bax with a decrease in Bcl-x_L_ expression and negates any involvement of Bak and Bcl-2 regulations in the damage induced by retinal ischemia 24 h after reperfusion. It also links caspase-3 to the deleterious effect induced by the elevation of IOP. Finally, although further investigations will be necessary to unravel the exact mechanism involved following I/R, this study highlights the importance of mitochondrial mediation of the apoptotic pathway in the deleterious effects in this model.
